# Does Taste Matter? How Anticipation of Cola Brands Influences Gustatory Processing in the Brain

**DOI:** 10.1371/journal.pone.0061569

**Published:** 2013-04-24

**Authors:** Simone Kühn, Jürgen Gallinat

**Affiliations:** 1 Clinic for Psychiatry and Psychotherapy, St. Hedwig-Krankenhaus, Charité University Medicine, Berlin, Germany; 2 The Center for Lifespan Psychology, Max Planck Institute for Human Development, Berlin, Germany; Duke University, United States of America

## Abstract

Brands surround us everywhere in daily life. Here we investigate the influences of brand cues on gustatory processing of the same beverage. Participants were led to believe that the brand that announced the administration of a Cola mixture provided correct information about the drink to come. We found stronger fMRI signal in right mOFC during weak compared to strong brand cues in a contrast of parametric modulation with subjective liking. When directly comparing the two strong brands cues, more activation in the right amygdala was found for Coca Cola cues compared with Pepsi Cola cues. During the taste phase the same beverage elicited stronger activation in left ventral striatum when it was previously announced by a strong compared with a weak brand. This effect was stronger in participants who drink Cola infrequently and might therefore point to a stronger reliance on brand cues in less experienced consumers. The present results reveal strong effects of brand labels on neural responses signalling reward.

## Introduction

Brands are ubiquitous phenomena in particular product brands such as Apple, McDonalds or Coca Cola surround us in our daily lives. Brands have been defined as names, terms, designs, symbols or any other features that identify one seller’s good or service as distinct from those of others [1]. Brands primarily consist of the sum of all mental associations that people have around it [2]. They are thought to influence perceptions and transform the experience of using products. In an early double-blind trial with branded analgesics, participants perceived the branded pain killer to be more effective than a chemically identical unbranded analgesic [3]. Brands are very powerful and have a high impact on people’s economic decisions, to the degree that consumers oftentimes prefer products of brands even among almost identical products [4]. Within the present study we set out to explore how brands are processed in the brain and how fictitious brand information can alter the perception and the neural correlates of product exposure.

To make economic choices between goods, the brain computes representations of their values. A great deal of research has been undertaken to determine the neural correlates of value representation in the human brain. Converging evidence from neuroscientific studies suggests that subjective value in decision-making is represented in the medial prefrontal cortex (mPFC) (including ventromedial prefrontal cortex (vmPFC) and medial orbitofrontal cortex (mOFC)). Activity within mPFC has been shown to be positively associated with the value assigned to various categories of products, suggesting that the brain encodes value in a “common currency” that allows for a shared valuation for different categories of goods [5]. Furthermore there is evidence that activation in the same areas represents the value of rewards even when choices are not required [6]. Additional causal evidence for the role of mPFC in coding of subjective value comes from lesion studies that have shown that patients with lesions in vmPFC are insensitive to future reward or punishment value in decision-making [7].

Another brain region that is strongly connected to mPFC [8], [9] and likewise sensitive for subjective value is the ventral striatum (including nucleus accumbens). A vast array of research implicates the importance of ventral striatum in reward-related processing [10], [11]. Striatal neurons code reward magnitude, incentive salience and fire more vigorously for preferred rewards [12].

In line with this research on subjective value previous neuromarketing research has shown that linguistic contextual information (“rich and delicious taste” vs. “monosodium glutamate”) has the potential to change the reported and experienced pleasantness of the identical delivered liquid food stimulus [13]. Differences were observed in pleasantness ratings as well as in an increased activity of the mOFC and ventral striatum during the “rich and delicious” word label. Similarly, Plassmann and colleagues showed that an announced increase in price of wine could likewise increase the reported pleasantness and neural processing of the same wine stimulus [14]. In a similar line a recent study has shown that the same photographs of foods with or without the widely known emblem for organic food in Germany were differently received by participants [15]. When foods were labeled as organic stronger brain activity in the ventral straitum was observed compared to the same food presented without the organic label.

In order to extend this research on value processing and its dependence on context factors such as verbal descriptions, price cues or organic labels, we set out to explore the influence of brand cues on the perception of soft drinks. For this purpose participants were told that they would receive one of four different Cola stimuli: one being the well known and strong brands Coca Cola and Pepsi Cola or the weak brand River Cola (generic brand sold by a big Germany supermarket chain) or a so called T-Cola that was introduced as a test drink mixed by a food science laboratory. Placed in the scanner participants consistently received the same Cola drink (a homogeneous equal mixture of the three sold brands). This was administered shortly after a brand cue was shown. The brand cue was explained to announce which of the soft drinks would be delivered in a few seconds. This experimental design allowed us to disentangle brain activity associated with the anticipation of a beverage of a certain brand and the actual taste processing of the administered mixture. We expected to find alterations of anticipatory as well as gustatory processing in brain regions that have been associated with subjective value such as mOFC and ventral striatum.

## Methods

### Participants

Fifteen healthy participants (age: mean = 31.4 years, ranging from 23 to 50; 7 female) participated on the basis of written informed consent. The study was conducted according to the Declaration of Helsinki, with ethical approval of the Deutsche Gesellschaft für Psychologie. All subjects had normal or corrected-to-normal vision. No subject had a history of neurological, major medical, or psychiatric disorder. All participants were right-handed.

### Behavioural Task

**Figure 1:**
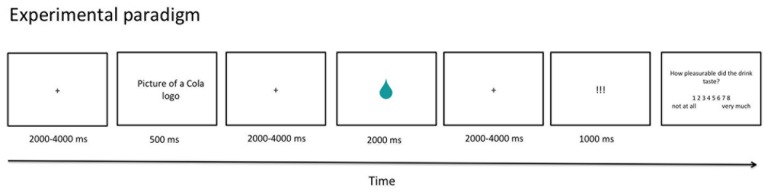
Schematic drawing of the paradigm. Pictures of the logos of Coca Cola, Pepsi Cola, River Cola and T-Cola were presented but cannot be depicted here due to creative common license of PLoS One.

During the experiment participants saw brand cues and got a beverage to taste administered via a tube system. The soft drink consisted to equal parts of Coca Cola, Pepsi Cola and River Cola (a generic brand sold in Germany). Participants were told that the brand cue would announce the soft drink they would actually receive shortly afterwards. Furthermore three real brands (Coca, Pepsi and River Cola) were introduced and participants were informed that another type of Cola that was mixed by a food science laboratory named T-Cola would be administered (*Figure 1*). The stimuli used as brand cues consisted of a picture depicting the logo, a bottle and a close up of the label on the bottle.

Each trial started with a variable oversampling interval of 2–4 s. Then a brand cue was shown for 500 ms announcing the drink to come – according to the cover story. As part of the cover story we showed four different injector syringes to the participants labelled with Coke, Pepsi, River and T-Cola before going into the scanner room. After the cue and another variable oversampling interval of 4–6 s a symbolic drop was shown for 2 s in the centre of the screen to announce the administration of 1 ml soft drink mixture via a perfusor that delivered the drink through a tube system with the ending piece being placed like a staw in the subjects’ mouth. The participants were instructed not to swallow until three exclamation marks appeared on the screen for 1 s after another variable oversampling interval of 4–6 s. The temporal separation of tasting and swallowing was employed to allow for separate modelling of the movement caused by swallowing. After each swallow phase participants were asked to rate how pleasurable the drink tasted on an 8-point rating scale ranging from 1 (“not at all”) to 8 (“very much”). Overall participants went through two blocks with 48 trials each, receiving 96 ml soft drink in total. The experiment had a total duration of 30–35 min.

After the imaging session participants went through a debriefing session in which we first asked participants to talk about their general impression of the experiment and second about the discriminability of the different drinks they have tasted. None of the subjects had the impression that the drinks were not discriminable. Some participants voiced strong preferences for certain beverages and dislike for others. At the end of the debriefing phase participants were informed that the beverage administered consisted of a mixture of the three brands and did not vary over trials and we explained the necessity of this deception to answer our research question.

### Scanning Procedure

Images were collected with a 3T Magnetom Trio MRI scanner system (Siemens Medical Systems, Erlangen, Germany) using a 12-channel radiofrequency head coil. First, high-resolution anatomical images were acquired using a T1-weighted 3D MPRAGE sequence (TR = 1900 ms, TE = 2.52 ms, TI = 900 ms, acquisition matrix = 256×256×176, sagittal FOV = 256 mm, flip angle = 9°, voxel size = 1×1×1 mm^3^). Functional images were collected using a T2*-weighted EPI sequence sensitive to BOLD contrast (TR = 2000 ms, TE = 30 ms, image matrix = 64×64, FOV = 192 mm, flip angle = 78°, voxel size 3×3×3 mm^3^, 33 axial slices). 480 image volumes aligned to AC-PC were acquired per run.

### fMRI Data Pre-processing and Main Analysis

The fMRI data were analysed using SPM8 software (Wellcome Department of Cognitive Neurology, London, UK). The first 4 volumes of all EPI series were excluded from the analysis to allow the magnetisation to approach a dynamic equilibrium. Data processing started with slice time correction and realignment of the EPI datasets. A mean image for all EPI volumes was created, to which individual volumes were spatially realigned by means of rigid body transformations. The structural image was co-registered with the mean image of the EPI series. Then the structural image was normalised to the Montreal Neurological Institute (MNI) template, and the normalisation parameters were applied to the EPI images to ensure an anatomically informed normalisation. A commonly applied filter of 8 mm FWHM (full-width at half maximum) was used. Low-frequency drifts in the time domain were removed by modelling the time series for each voxel by a set of discrete cosine functions to which a cut-off of 128 s was applied. The statistical analyses were performed using the general linear model (GLM). We modelled the brand cue, the symbolic drop during tasting and the swallowing sign as an event. These vectors were convolved with a canonical hemodynamic response function (HRF) and its temporal derivatives to form regressors in a design matrix. For the parametric modulation analysis we included the subjective taste judgement at the end of each trial as a parametric modulator into the design matrix of each individual subject. The parameters of the ensuing general linear model were estimated and used to form contrasts, testing for main effects and interactions. The resulting contrast images were then entered into a series of one sample T-tests at the second (between subject) level. This is the usual summary statistic approach to random effects analyses. For display purposes the resulting SPMs were thresholded at *p*<0.001 (z >3.09, uncorrected) and a significant effect was reported when the volume of the cluster was greater than the Monte Carlo simulation determined minimum cluster size above which the probability of type I error was below 0.05 (AlphaSim, [35]). The resulting maps were overlaid onto a normalized T1 weighted MNI template (colin27) and the coordinates reported correspond to the MNI coordinate system.

For the signal change analysis we used clusters of interest as ROIs. In order to explore effects of brand cues we extracted per cent signal change for each subject, region and condition over a time window of 4–6 s after stimulus onset (http://marsbar.sourceforge.net/, [36]).

## Results

### Pleasantness Ratings

**Figure 2:**
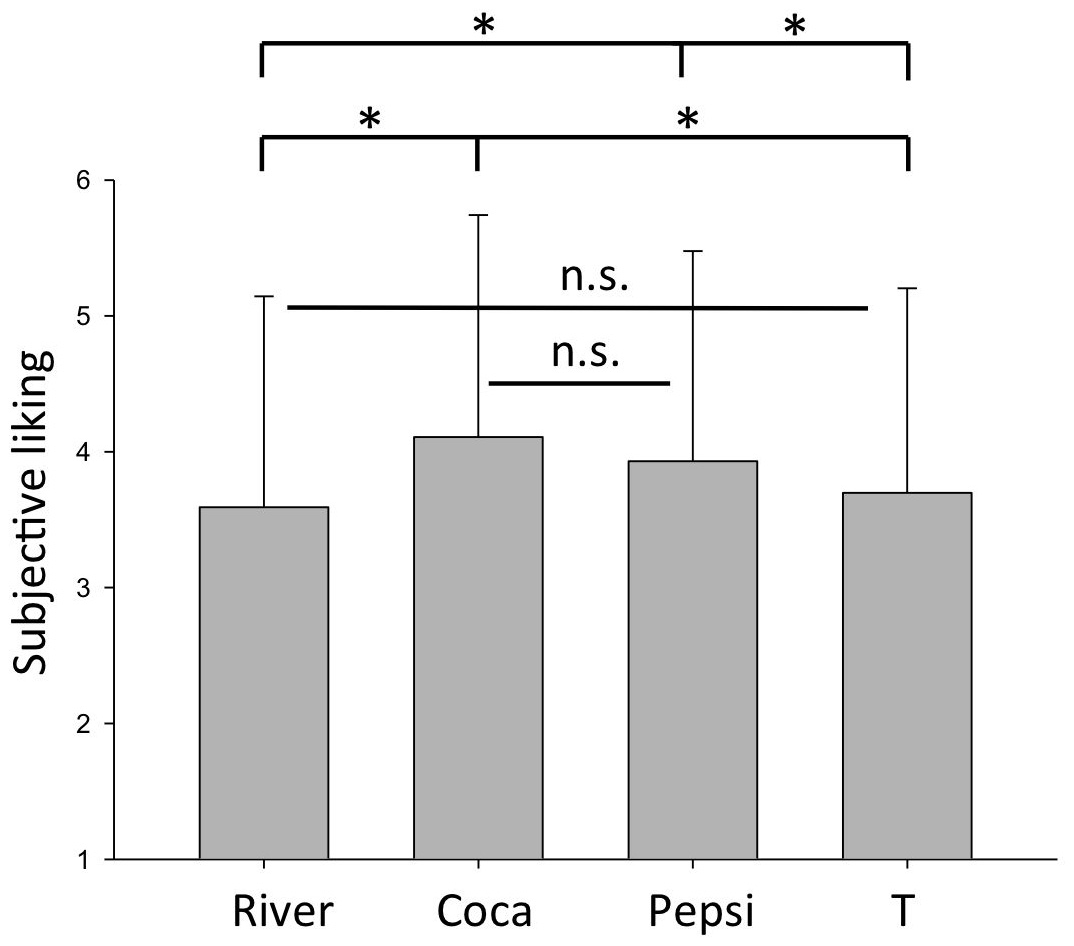
Liking judgement of Cola taste stimulus administered during scanning split up according to the different brand cues. Error bars depict the standard deviation.

We found a significant effect of cue brand on the rating of liking (*F*(3,42) = 6.871; *p*<0.05). Participants rated the beverage announced as being Coca Cola significantly more likable compared to River Cola (*t*(14) = 3.507, *p*<0.01) and T-Cola (*t*(14) = 3.034, *p*<0.01). Furthermore a significant difference in pleasure rating was found between Pepsi Cola and River Cola (*t*(14) = 2.934, *p*<0.05) as well as T-Cola (*t*(14) = 2.342, *p*<0.05). In contrast participants reports did not differ significantly between Coca Cola and Pepsi Cola (*t*(14) = 0.875, *p*<0.396) nor between River Cola and T-Cola (*t*(14) = 1.091, *p*<0.294) (*Figure 2*).

### Brand Cue Processing

**Figure 3:**
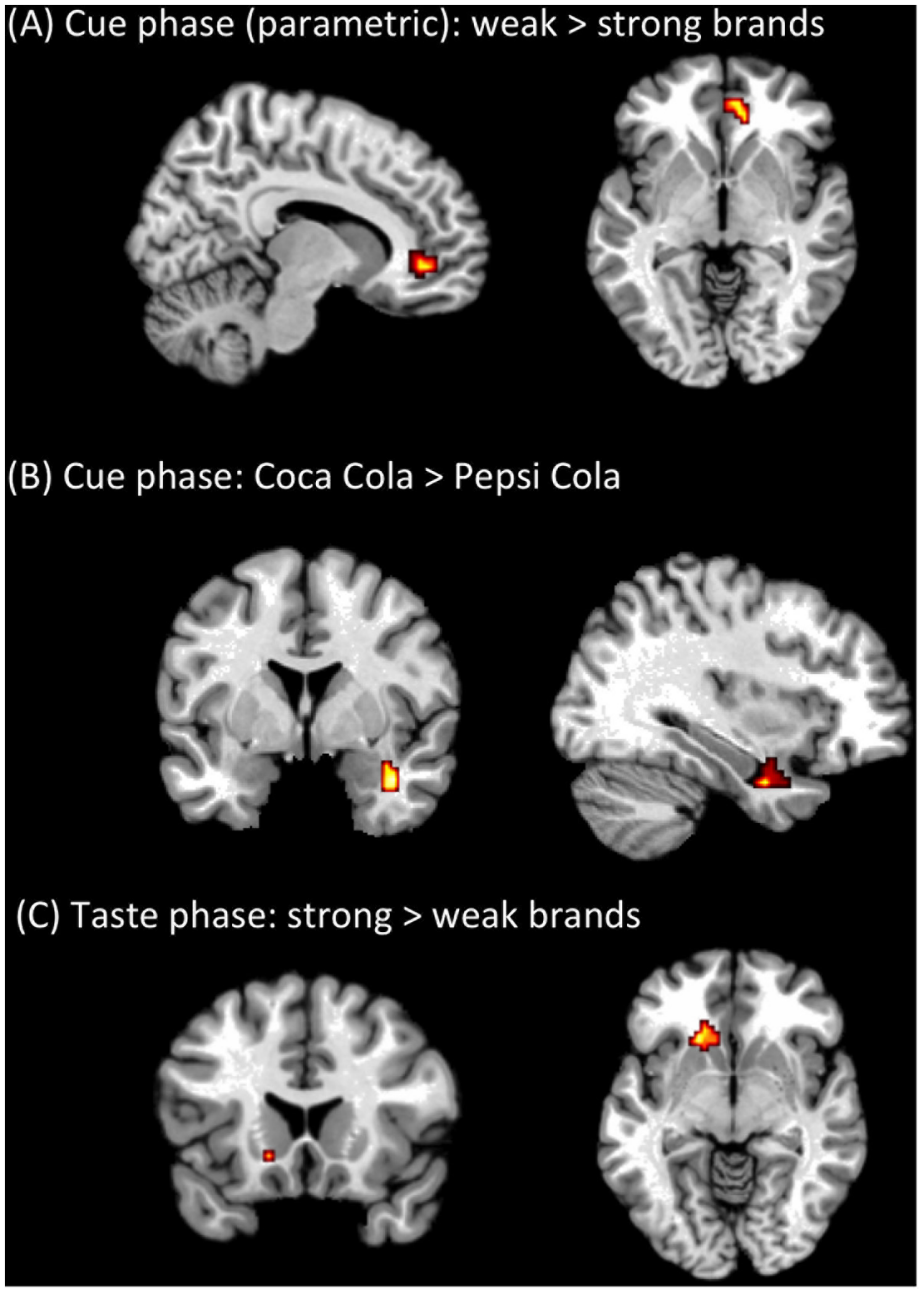
Contrast maps depicting (A) the contrast of weak (River Cola, T Cola) vs. strong (Coca Cola, Pepsi Cola) brands during cue presentation parametrically modulated with liking judgement over 15 subjects (p<0.001, cluster >18) mapped onto an MNI template shows activity within medial orbitofrontal cortex (mOFC: 9, 42, −6, BA 10). (B) Coca Cola vs. Pepsi Cola brand during cue phase shows activity within right amygdala (39, 0, −27). (C) Contrast of strong (Coca Cola, Pepsi Cola) vs. weak (River Cola, T Cola) brands during taste phase shows activity within left striatum (−15, 27, −9).

When comparing neural processing during brand cue perception of a strong (Coca Cola, Pepsi Cola) with a weak brand (River Cola, T Cola) we found no significant clusters of activation. When weighing each trial with the individual pleasantness judgement reported at the end of each trial (parametric modulation) we found a significant cluster in right mOFC (MNI coordinates: 9, 42, −6, BA 10) for the contrast weak vs. strong brand cues (*Figure 3A*). This indicates that the liking judgement more strongly covaried with mOFC activation if the brand cue itself was not very strong or informative. One may argue that River Cola can be considered a brand although this generic brand is not subject to advertisement in Germany. In contrast participants can have no associations with T Cola since this brand has been invented by the experimenters. In order to test whether the grouping of River Cola and T Cola in our analysis was justified we compared the corresponding beta values and found no significant difference between both brand cues (*t*(14) = 0.368, *p* = 0.72), which may seen as support for pooling them under the label of weak brands. However, when excluding T-Cola and contrasting the parametrically modulated River Cola with Coca and Pepsi Cola we likewise found activation in the mOFC.

A comparison between the parametrically modulated mOFC signal between participants who reported to drink no Cola (n = 6) and participants who reported to drink Cola at least once during a regular week (n = 9) did not reveal any difference (*p*>0.56).

Additionally, we computed the direct contrast between brand cue processing of Coca Cola and Pepsi Cola. We found significantly stronger activation within the right amygdala (39 0 −27, *Figure 3B*) when Coca Cola brand cues were compared with Pepsi Cola cues and no significant clusters was found for the reverse contrast or during the taste phase.

### Taste Processing

**Figure 4:**
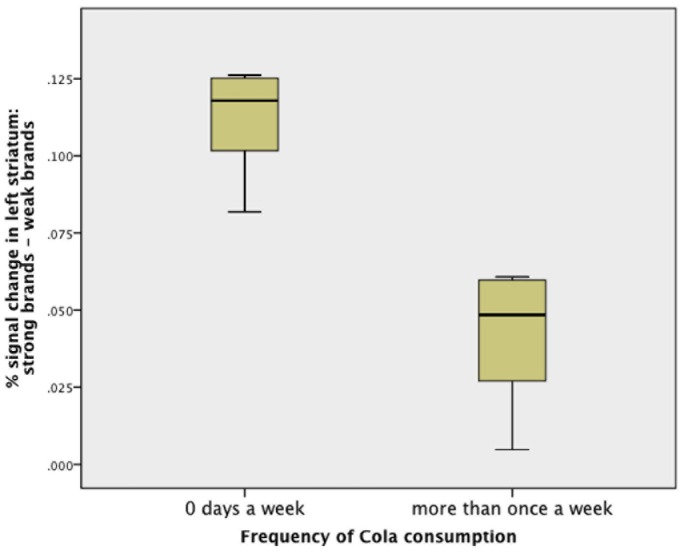
Box plot depicting the difference in BOLD per cent signal change in left striatum in strong (Coca Cola, Pepsi Cola) compared to weak (River Cola, T Cola) brands in participants who report to drink Cola with a frequency of 0 days a week on average and participants reporting to report Cola more than once a week. Error bars depict the standard deviation.

When focussing on brain regions showing brand effects during the taste phase we found significantly more activity in the left ventral striatum (−15, 27, −9) during tasting the beverage when it was announced by means of cuing with a strong brand compared with a weak brand (*Figure 3C*). We extracted BOLD per cent signal changes from left ventral striatum and compared the signal change between tasting strong vs. weak brands. BOLD signal during strong brands was significantly different from zero (Coca Cola: t(14) = 2.34, p<0.05, Pepsi Cola: t(14) = 2.17, p<0.05). We found a significant difference between participants who reported to drink no Cola and participants who reported to drink Cola at least once during a regular week (*Figure 4*). Participants who report not to drink Cola show a stronger ventral striatum effect in response to strong vs. weak brands during tasting.

When parametrically modulating the taste phase BOLD signal with the subjective judgement no clusters reach significance for the comparison of strong and weak brands.

BOLD signal in mOFC during the brand cue phase was not significantly associated with BOLD signal in the ventral striatum during the taste phase.

## Discussion

Within the scope of the present study we investigated the influence of Cola brand cues on the neural correlates of gustatory processing of the ever same soft drink mixture administered shortly after the brand cue in an fMRI scanner. Brand cues did significantly affect the later self-reported pleasantness ratings. The strong brands Coca and Pepsi Cola received higher pleasantness ratings compared to the weak brands River and T-Cola. We did not find a significant difference between Coca Cola and Pepsi Cola preference. The so-called “Pepsi Paradox” states that people exhibit a reliable preference for Coca Cola when brand information is available (e.g. in the supermarket), but no reliable preference for Coke when no brand information is available (e.g. in blind taste tests) [16]. Accordingly, one might therefore have predicted a preference for the soft drink mixture when it was announced as Coca Cola compared with Pepsi Cola, based to the so-called “Pepsi Paradox”. But without a reference to a blind taste condition to compare the results to, it is difficult to conclude that the present findings are not in line with the “Pepsi Paradox”. It may still be the case that also in the present sample Pepsi Cola would have been judged as more pleasant than Coca Cola in a blind test.

We set out to compare strong and weak brands. We pooled River Cola and T Cola as weak brands although participants may have preexisting associations with River Cola but not with the invented T-Cola. We did that mostly because River Cola is a generic brand that is not subject to advertisement, and we strongly doubt that German customers would be able to recall the logo of River Cola when prompted to. When comparing brain activation between strong and weak brands during the cue phase, weighted according to the preference rating after each trial, we found stronger activation in right mOFC during weak compared with strong brands. Previous human fMRI studies have placed individuals in simple choice situations and found that BOLD activity in the mOFC correlates with behavioral measures of stimulus values [17], [18]. These findings are consistent with monkey neurophysiology studies that have found stimulus value coding in OFC neurons during choice tasks [19], [20]. Based on this view of mOFC the present findings may indicate that participants rely on stimulus values encoded in mOFC more strongly whenever weak brands do not offer sufficient guidance to decide about the drinks pleasantness. When on the other hand a strong brand is expected, this strong brand cue overrides elaborate processing of stimulus value in mOFC, since the brand is well known and its associations can be easily retrieved without an additional assessment of the subjective stimulus value. That mOFC can predict consumer choice has been nicely demonstrated in a pattern classification study in which preferences for cars were inferred from activity of mOFC [21]. A previous study on soft drinks administered pure Coca Cola and Pepsi Cola in an anonymous and a real cue condition [16]. During the anonymous tasting mOFC activity was positively correlated with the subjects’ reported preference for the beverages. This finding reveals that mOFC is related to taste preferences when no brand cue is delivered. Consequently one may interpret the present finding as an indication that the announcement by means of weak brands has a similar effect as the absence of brand cues. Therewith the present study extends previous literature on the influence of linguistic contextual information [13] and pricing [14] that has shown a positive association between activity in mOFC and positive linguistic information as well as information on the product price. Strong brands in contrast to high price and positive linguistic information seem to attenuate stimulus value related to mOFC stimulus value processing that is present when weak brands are announced. But this difference in results could well be due to the fact that the present design allowed us to disentangle cue and taste related processing, whereas the previous studies delivered the linguistic and pricing information in synchrony with the taste stimulus.

In a direct comparison of Coca Cola and Pepsi Cola during the cue phase, we found significantly more activation in right amygdala associated with the Coca Cola cue. The amygdala is generally known for its role in emotion processing. Traditionally it has been linked to negative emotions [29], in particular to fear [30]. But more and more evidence exists that questions this strong distinction on a valence dimension [31]. Furthermore the amygdala has been found to respond to the prediction of monetary reward [32]. A recent study has addressed the question whether amygdala is sensitive to valence and/or intensity [33]. It was found that OFC responds to valence, whereas amygdala responds to intensity of stimuli [34]. Accordingly, Coca Cola cues elicit more intensity processing in the amygdala compared to Pepsi Cola, which may contribute to Pepsi being the eternal runner-up.

When focusing on the neural differences between gustatory processing of identical beverages when a strong compared to a weak brand was announced we found significantly more activation in the left ventral striatum when a strong brand was expected. The ventral striatum primarily consists of the nucleus accumbens and receives extensive projections from orbitofrontal, ventromedial, and ventrolateral cortex and dopaminergic input from ventral tegmental area [22]. In rats the ventral striatum has been described as the “hedonic hot spots” since drug microinjections of opioids amplify the liking of sweet taste rewards [23], [24]. In humans the ventral striatum has been found to be activated when reward is received [25] as well as when reward is expected. In contrast to mOFC that codes stimulus value, some authors have argued that ventral striatum codes mainly saliency [26], [27]. Salience can be defined as a property of stimuli that are both unexpected and elicit an attentional–behavioural switch. In a previous study the ventral striatum has been shown to be only active when money could be gained by acting in a specific way but not when money was gained passively [26]. This points to the fact that reward processing is to be understood in the context of motivated behaviour. In a previous neuromarketing study the ventral striatum has been associated with product preference whereas the mOFC was observed to be active prior to the purchase decision [28]. Furthermore, the association between strong brand cues and enhanced signal in the ventral striatum can be seen in light of the previously mentioned study that presented identical pictures of food with or without the emblem for organic food and found stronger ventral striatum activation during organic labels [15].

The post-hoc finding that the signal difference in the ventral striatum during taste phase between the drinks announced as strong compared with weak brands is bigger in participants who do not drink Cola frequently fits to the notion that striatum signal is relevant for behavior. One may speculate that participants who drink Cola less frequently perceive the strong brand cues as more salient because they have less prior experience with Cola.

### Conclusions

To summarize, his study is the first to test the effects of brand cues onto gustatory processing of the same beverage. We found a clear preference for the same beverage when it was believed to be Coca Cola or Pepsi Cola (strong brands) compared with River Cola or T-Cola (weak brands) in self-reported pleasantness ratings. In the fMRI data we found stronger signal in mOFC parametrically modulated with pleasantness ratings during weak as compared to strong brand cues. This potentially indicates a stronger reliance on stimulus value processing when the brand cue is less informative. Furthermore stronger activation in the right amygdala was found for Coca Cola cues compared with Pepsi Cola cues. During the taste phase the same soft drink elicited stronger activation in left ventral striatum when it was previously announced as a strong brand compared with a weak brand. This effect was stronger in participants who drink Cola very infrequently and might point to a bigger reliance on brand cues in less experienced consumers. Taken together the present results show the strong effects of brand cues on self-reported pleasantness as well as on neural responses signalling reward in the brain.
